# Insight into Antioxidant Activity and Peptide Profile of Jinhua Ham Broth Peptides at Different Cooking Times

**DOI:** 10.3390/antiox12030606

**Published:** 2023-03-01

**Authors:** Ziyi Yang, Jiaming Cai, Evans Frimpong Boateng, Lujuan Xing, Wangang Zhang

**Affiliations:** Key Laboratory of Meat Processing and Quality Control, Ministry of Education China, Jiangsu Collaborative Innovation Center of Meat Production and Processing, Quality and Safety, College of Food Science and Technology, Nanjing Agricultural University, Nanjing 210095, China

**Keywords:** Jinhua ham, broth, bioactive peptides, antioxidant activity, peptide profile

## Abstract

This present study aimed to investigate the effects of various cooking times (1 h, 1.5 h, 2 h, 2.5 h, named as JHBP-1, JHBP-1.5, JHBP-2, JHBP-2.5) on the antioxidant activity and peptide profile of Jinhua ham broth peptides (JHBP). The peptides extracted from uncooked ham were used as an uncooked group with the name of JHBP-0. The results revealed that the antioxidant efficacy in the four cooked groups changed dramatically compared to JHBP-0. After cooking, the DPPH radical scavenging activity, hydroxyl radical scavenging activity and superoxide anion radical scavenging activity decreased, except for the Fe^2+^ chelation and ABTS^+^ scavenging capacity which increased significantly. However, the cooked groups still showed a strong antioxidant capacity. In particular, the superoxide anion radical scavenging ability and the Fe^2+^ chelation action were significantly stronger compared to glutathione (GSH) and butylated hydroxytoluene (BHT) (*p* < 0.05). JHBP-1.5 also displayed stronger antioxidant capacity than the other three cooked groups, and its secondary structure and mass distribution changed significantly after cooking, specifically with an increased proportion of helix and <1 kDa peptides. Moreover, the constitution of free amino acids (FAAs) and the types of peptides released in the broth increased significantly with a longer cooking time. In total, 1306 (JHBP-0), 1352 (JHBP-1), 1431 (JHBP-1.5), 1500 (JHBP-2), and 1556 (JHBP-2.5) peptide sequences were detected using LC-MC/MC. The proportion of <1 kDa peptides also gradually increased as the cooking time extended, which is consistent with the molecular weight distribution measurements.

## 1. Introduction

Dry-cured ham is a premium product with a long history, and the production process could last for 6–24 months [1]. During the long processing procedure, dry-cured ham undergoes many physical and chemical changes, such as structural and textural changes, protein hydrolysis, and lipid oxidation, etc. [2]. By the action of endogenous proteolytic enzymes, proteins are broken down into peptides and free amino acids (FAAs) [3]. The peptides produced in decomposition are often biologically active and beneficial for human health [4]. Numerous reports have demonstrated that Chinese dry-cured ham, including Jinhua and Xuanwei, and Spanish dry-cured ham possess rich antioxidant peptides [5,6].

It is also worth noting that the traditional Chinese dry-cured ham has a salt concentration of 6–15%, which is substantially higher than dry-cured ham from European countries [7]. Because of its high salt content, local Chinese more commonly use Jinhua ham to prepare broth rather than consume it directly. Particularly in some southern provinces of China, there is the tradition of preparing broth with pork, ham, or other meat because of their unique flavor and rich nutrition. Dry-cured ham broth is often cooked without other ingredients, and in addition to being a traditional delicacy, it is also documented in ancient Chinese medical books as a medicinal dish with the ability to heal wounds and relieve pain [8]. During heating, the peptides and amino acids can be released into the broth. Cooking also leads to more thorough protein breakdown, which in turn produces more peptides and amino acids, and different cooking temperatures and durations might induce varying outcomes [9]. Although cooking can increase the content of peptides, heating can also change the secondary structure of peptides, which could regulate the antioxidant activity of peptides [10].

Our previous works have revealed the presence of antioxidant peptides in both Jinhua ham and Xuanwei ham [11,12]. However, the generation and the release of bioactive antioxidant peptides from dry-cured ham during cooking have not been reported. Therefore, this study aimed to examine the effects of various cooking times on the quantity of FAAs and small molecular peptides in Jinhua ham broth, their antioxidant properties, and peptide profiles by simulating traditional soup-making conditions. Findings from this research highlight the changes of bioactive peptides in Jinhua ham broth during cooking, and provide suggestions for selecting the optimum cooking time for Jinhua ham broth.

## 2. Materials and Methods

### 2.1. Materials

Jinhua hams were secured from Jinzi Ham Food Company (Jinhua, Zhejiang, China). Hemin was purchased from Wako Chemicals USA (Richmond, VA, USA). o-Phthalaldehyde (OPA, 99% purity) and tyrosine (≥99%) were secured from J&K Scientific (Beijing, China). 1,1-Diphenyl-2-picrylhydrazyl (DPPH) was sourced from Coolaber Technology Co., Ltd. (Beijing, China). Ferrozine (98% purity) and bovine serum albumin (BSA) were purchased from Solarbio Biotechnology Co., Ltd. (Beijing, China). Aprotinin (titer > 3 TIU/mg) and bacitracin (titer > 60 Units/mg) were bought from Macklin Biochemical Co., Ltd. (Shanghai, China). BHT (>99% purity), lysozyme (≥20,000 U/mg), and formic acid (≥98%, HPLC) were acquired from Aladdin Biochemical Technology Co., Ltd. (Shanghai, China). Ferric-reducing antioxidant power kits, 2,2′-azino-bis (3-ethylbenzothiazoline-6-sulfonic acid) (ABTS^+^) radical scavenging capacity kits, and glutathione (GSH) were purchased from Beyotime Biotechnology Co., Ltd. (Shanghai, China). Hydroxyl and superoxide anion-free radical scavenging activity kits were sourced from Nanjing Jiancheng Bioengineering Institute (Nanjing, China). Other analytical-grade reagents were purchased from Mall-bio-Biotechnology Co., Ltd. (Nanjing, China). Phe-Ser-Gly-Leu (98% purity) was synthesized by Qiangyao Biotechnology Co., Ltd. (Wuhan, China).

### 2.2. Sample Preparation

Our methodology was adapted from Xing et al. [13] with some modifications. Six hams were selected randomly, noting that Jinhua hams usually require a 12-month ripening period with a traditional fermentation process. The middle segment of the muscle biceps femoris was taken for subsequent experiments. We divided each of the six hams evenly into five portions (each weighing 25 g), and took out one portion as the uncooked group (JHBP-0). The uncooked group samples were placed in a 500 mL centrifuge bottle after 25 g of uncooked Jinhua ham was ground with a meat grinder (JZR-G608, Zhongshan Jinzheng Electrical Appliance Co., Ltd., Zhongshan, China) in 100 mL of hydrochloric acid (0.01 mol/L, pH = 2) and homogenized four times at 16,000 rpm/min for 10 s. The specimen was placed at 4 °C for 2 h and then centrifuged (12,000× *g* at 4 °C/30 min), and the supernatant was filtered through two layers of gauze and added to 3 times the volume of 40% (*v/v*) of ethanol. The samples were left at 4 °C for 12 h before being centrifuged at 12,000× *g* for 20 min at 4 °C to remove the protein. After the ethanol was removed by spin evaporation, the sample was freeze-dried, and the dried sample was stored at −20 °C.

Four cooked groups (JHBP-1, JHBP-1.5, JHBP-2, JHBP-2.5) were pretreated to extract peptides from the broth. We then took the other four portions among the selected six hams for subsequent experiments. After removing the fat and muscle tendon from the Jinhua ham, the muscle was sliced into 0.3 × 0.3 × 0.3 cm pieces. Here, we referenced Erickson’s methodology [14] for defatting with some modifications. Dichloromethane and methanol 2:1 (*v/v*) were mixed, the meat blocks were soaked at a ratio of 3:1 (*v/m*), and the fat was removed after 3 min of soaking, which was repeated three times. The meat was dehydrated in an oven at 45 °C for 0.5 h and then cooked according to the design of Zhang et al. [15] with minor modifications. After that, the meat was added to water at a ratio of 1:4 (*w/v*), and the water was boiled in an induction cooker at a cooking power of 2100 W. The power was then adjusted to 300 W and maintained for 1 h, 1.5 h, 2 h, and 2.5 h. The broth was filtered through a double layer of gauze and then cooled naturally at room temperature. Then, 100 mL of broth and three times the volume of 40% (*v/v*) ethanol were mixed and left for 12 h at 4 °C. After centrifuging at 12,000× *g* for 20 min at 4 °C to remove the protein, samples were freeze-dried after the ethanol was removed by spin evaporation, and the dried sample was stored at −20 °C.

### 2.3. FAAs Analysis

The 1 mL of the mixed broths in the uncooked group and four cooked groups were adulterated with Milli-Q water to 50 mL, and 0.5 mL of the diluents were homogenized with 2 mL 5% (*w/v*) sulfosalicylic acid. After standing at 4 °C for 0.5 h, the samples were centrifuged at 12,000× *g*/min for 20 min to remove proteins and impurities. The afloat was then taken out to evaluate the free amino acid content using the amino acid analyzer (L-8900, Hitachi Company, Tokyo, Japan).

### 2.4. Peptide Content Determination

The peptide content of the samples was estimated according to a modified procedure of Xing et al. [11]. The 200 mg of OPA was solvated in 5 mL of methanol, and then 125 mL of 0.1 M sodium tetraborate, 12.5 mL of 20% (*w/w*) sodium dodecyl sulfate, and 500 µL of β-mercaptoethanol were added and fixed to 250 mL with a Milli-Q aqueous solution. The OPA mixture was prepared temporarily before use. Then, the 100 µL of the extracts (dissolved in Milli-Q water, 1 mg/mL) were mixed with 2 mL of OPA solutions and incubated for 2 min at room temperature without light. The absorbance of the mixture was measured at 340 nm by UV spectrophotometer (Spark2010014158, TECAN Austria GmbH, Grödig, Austria), and a linear concentration was determined using casein as a standard. The peptide constituent was determined depending on the standard curve.

### 2.5. Determination of Peptide Molecular Weight (MW) Distribution

An FPLC system (UPC10, GE Healthcare, Danderyd, Sweden) was used to separate the peptides. Before analysis, freeze-dried samples were disintegrated in Milli-Q water to 5 mg/mL and sieved by the membrane (0.22 µm, Solarbio Biotechnology Co., Ltd., Beijing, China). The segregation was followed with the procedure of Zhao et al. [16]. In the analytical runs, the SuperdexTM peptide 10/300 GL (GE Healthcare, Danderyd, Sweden) was employed. Each elution was performed with 35 mL PBS (50 mM, 100 mM NaCl, pH 7.0) at a flow rate of 0.75 mL/min. For detection, the injection mass was 500 µL, and the absorbance at 280 nm. BSA (66.446 kDa), lysozyme (14.306 kDa), aprotinin (6.511 kDa), bacitracin (1.422 kDa), hemin (0.652 kDa), Phe-Ser-Gly-Leu (0.423 kDa), and tyrosine (0.181 kDa) were used for mass calibration.

### 2.6. DPPH Free Radical Scavenging Ability

The DPPH scavenging efficacy of the peptides was evaluated following the methods of Xing et al. [12]. GSH and BHT were prepared as the positive control, and except that BHT was dissolved with methanol, other groups were dissolved with Milli-Q water to prepare the sample concentrations of 0.5, 1, 2, 3, 4, and 5 mg/mL. The 1 mL of sample solution was fused with 1 mL of DPPH (0.2 mmol/L, dissolved in methanol). The 1 mL of Milli-Q water was added to 1 mL of methanol as a blank batch and 1 mL of 0.2 mmol/L DPPH was mixed with 1 mL of methanol as a control. All the mixtures were incubated at room temperature for 30 min while shielded from light. At 517 nm the absorbance of the mixture was measured. The scavenging activities of JHBP, GSH, and BHT were calculated following the equation below:

$$\mathrm{DPPH}\;\mathrm{radical}\;\mathrm{scavenging}\;\mathrm{activity}\;(\%)=[\left(A_{con}-A_{sam}\right)/\left(A_{con}-A_{bla}\right)]\times 100$$

*A_sam_* indicates the absorbance values of sample solution at 517 nm; *A_con_* indicates the absorbance values of methanol replace sample solution at 517 nm; *A_bla_* indicates the absorbance values of Milli-Q water:methanol (1:1) at 517 nm;

### 2.7. Hydroxyl Radical Scavenging Efficacy (•OH)

BHT was dissolved with methanol, and other groups were dissolved in Milli-Q water to prepare the concentrations of 0.5, 1, 2, 3, 4 and 5 mg/mL, respectively. The 0.5 mL peptide mixture was fused with 0.2 mL of substrate application solution and 0.4 mL of application solution, which was then warmed at 37 °C for 1 min. After that, 2 mL of the color developer (Griess reagent) was added and kept for 20 min at ambient conditions. The 0.5 mL of Milli-Q water was used to substitute the peptide solution as the control group, and 0.7 mL of Milli-Q water was utilized as the peptide solution, and substrate application solution as the blank group. The absorbance value was computed at 550 nm. The scavenging activities of JHBP, GSH, and BHT were quantified based on the following equation:

$$\mathrm{Hydroxyl}\;\mathrm{radical}\;\mathrm{scavenging}\;\mathrm{activity}\;(\%)=[\left(A_{con}-A_{sam}\right)/\left(A_{con}-A_{bla}\right)]\times 100$$

*A_sam_* indicates the absorbance values of sample solution at 550 nm: *A_con_* indicates the absorbance values of Milli-Q water replace sample solution at 550 nm: *A_bla_* indicates the absorbance values of Milli-Q water replace sample solution and substrate application at 550 nm.

### 2.8. Superoxide Anion Radical Scavenging Action (SOSA)

The SOSA assay kits were used for SOSA assessment. BHT was dissolved with methanol, and other groups were dissolved in Milli-Q aqueous solution. The concentrations were 0.5, 1, 2, 3, 4 and 5 mg/mL, respectively. The 0.2 mL of peptide solution was vortexed with 2 mL of working solution and incubated at 37 °C for 40 min. After that, 2 mL of the developer was added to the samples, and samples were left for 10 min at room temperature. The Milli-Q water was used to substitute the peptide solution as the control group and the working solution as the blank group. The absorbance value was measured at 550 nm. The scavenging activities of JHBP, GSH, and BHT were calculated by adopting the equation below:

$$\mathrm{Superoxide}\;\mathrm{anion}\;\mathrm{radical}\;\mathrm{scavenging}\;\mathrm{activity}\;(\%)=[\left(A_{con}-A_{sam}\right)/\left(A_{con}-A_{bla}\right)]\times 100$$

*A_sam_* indicates the absorbance values of sample solution at 550 nm; *A_con_* indicates the absorbance values of Milli-Q water replace sample solution at 550 nm; *A_bla_* indicates the absorbance values of working solution at 550 nm.

### 2.9. Fe^2+^ Chelating Capacity

Ferrous ion chelation experiments followed the study of Chen et al. [17]. The 1 mL of the mixture (1, 2, 3, 4, 5 mg/mL) was vortexed thoroughly with 0.05 mL of FeCl_2_ (2 mmol/L), and afterward the 0.2 mL of ferrozine (5 mmol/L) was added to activate the reaction. The Milli-Q water was substituted for the peptide mixture as the control group, and Milli-Q water was used as the blank group. The absorbance at 562 nm was measured after 10 min incubation at room temperature. The scavenging activities of JHBP, GSH, and BHT were estimated by the following equation:

$${\mathrm{Fe}}^{2+}\;\mathrm{chelating}\;\mathrm{activity}\;(\%)=[\left(A_{con}-A_{sam}\right)/\left(A_{con}-A_{bla}\right)]\times 100$$

*A_sam_* indicates the absorbance values of sample solution at 550 nm; *A_co_* indicates the absorbance values of Milli-Q water replace sample solution at 550 nm; *A_bla_* indicates the absorbance values of Milli-Q water at 550 nm.

### 2.10. Ferric-Reducing Antioxidant Power (FRAP)

The technique of Yu et al. [18] was used to perform the measurement with some modifications. The 5 µL of peptide solutions (1 mg/mL, dissolved in PBS) were mixed with 180 µL of FRAP operational mixture, and the optical density value was determined at 593 nm after incubation at 37 °C for 5 min. Concentration gradients of 0, 9.375, 18.75, 37.5, and 75.0 µM FeSO_4_ were configured instead of peptide solution, and the absorbance value was calculated to plot the standard curve. The peptide solution was replaced by water as a blank control. The FeSO_4_ equivalent antioxidant capacity of JHBP was presented as µmol Fe^2+^/g.

### 2.11. ABTS^+^ Radical Scavenging Capacity

For the ABTS^+^ method, the antioxidant capacity of the species was shown as Trolox-Equivalent Antioxidant Capacity (TEAC). The 10 µL of peptide solutions (1 mg/mL, dissolved in PBS) were fused with 200 µL of ABTS^+^ working mixture, and the optical value was estimated at 734 nm by incubation at room condition for 4 min. Concentration gradients of 0, 37.5, 75.0, 125, 250, 500, and 750 µM Trolox were configured instead of peptide solution, and the absorbance value was measured to plot the standard curve. The peptide mixture was replaced with water as a blank control. The TEAC of JHBP was expressed as µmol TE/g.

### 2.12. CD Spectra of the Peptides

The secondary structure of peptides was detected by the CD spectrum (J-1500, JASCO, Tokyo, Japan). The peptides were dissolved in the Milli-Q water with a concentration of 100 μg/mL. The specific parameters were as follows, and the circular dichroism chromatograph was 0.1 cm with a recording wavelength span of 190–250 nm. The scanning velocity was 50 nm/min with a bandwidth of 1.0 nm as well as a resolution of 0.5 nm. The test was carried out at room temperature. Each peptide was scanned three times.

### 2.13. Characterization of Peptide Sequences

The desalted peptides were diffused in 0.1% formic acid (solvent A) before being analyzed by Nano LC system (EASY-nLC 1200, Thermo Scientific, Waltham, MA, USA) coupled with Orbitrap Exploris 480 mass spectrometer with FAIMS (High-Field Asymmetric Waveform Ion Mobility Spectrometry). The analytical columns (Acclaim PepMap^®^ RSLC, C18, 75 μm × 15 cm, 3 μm, 100 Å, Thermo Scientific, Waltham, MA, USA) were applied to perform the chromatographic separation using a 30-min linear gradient of 3–35% buffer B (80% acetonitrile with 0.1% FA) at a flow rate of 0.3 µL/min. FAIMS had a compensation voltage (CV) of −45 V and −65 V. The mass spectrometer was set to a data-dependent analysis (DDA) mode with a dynamic exclusion of 30 s and full-scan MS spectra (*m/z* 350–1500) with a resolution of 60,000 (*m/z* 200) and a resolution of 15,000 (*m/z* 200) in MS/MS scans.

The natural peptides extracted from broths were identified using PEAKS Studio X Pro (Version 10.6). The UniProt database was used to identify peptides and proteins of origin, with a parent mass error tolerance of 10 ppm and a fragment mass error tolerance of 0.2 Da. Proteomes from Sus scrofa (Pig) and no enzymes were chosen. For peptide sequences, the processed data used database searching with an FDR ≤ 1%.

### 2.14. Statistical Analysis

The data were analyzed by SAS software (Cary, SAS Company, NC, USA) depending on the one-way analysis of variance and Duncan’s multiple range test. When *p* < 0.05, it was considered a significant difference. The results were presented as mean ± standard error.

## 3. Results and Discussion

### 3.1. FAAs

As indicated in Table 1, the FAA content of crude peptides increased with increased cooking time (*p* < 0.05). The FAA concentration in the 2.5 h cooking group samples was 1213.86 mg/100 mL broth, which was substantially higher compared to the other groups (*p* < 0.05). The concentrations of Lys, Glu, Ala, Leu, Arg, and Val were much higher in all five experimental groups than in other AAs. In contrast, the concentrations of Met and His were the lowest in all groups.

The release of soluble substances was reported to be affected by the temperature and cooking time of pork [19,20]. Glu, total FAAs, and hypoxanthine nucleotide (IMP) were released from the muscles into the broth during cooking and their levels were gradually increased. When the chicken broth was heated at 80 °C and 90 °C for 50 and 60 min, the non-protein nitrogen and total FAAs were also increased [21]. All of the above findings imply that cooking could influence the release of FAA of meat and meat products.

### 3.2. Peptide Content in Jinhua Ham Broth

As shown in Table 2, the cooking process enhanced the content of peptide extracts. The longer the cooking time, the more peptide extracts were obtained. The broth from Jinhua ham contained 15.94 g of crude extracts per 100 g ham after 2.5 h of cooking. The crude extracts obtained from JHBP-1, JHBP-1.5, JHBP-2, and JHBP-2.5 were substantially higher (*p* < 0.05) compared to the control (9.77 g/100 g ham). No significant variation (*p* > 0.05) existed between cooking treatments of JHBP-2 and JHBP-2.5. In contrast, the final extracted peptide increased, and JHBP-2.5 had the highest peptide extraction of 13.85%. The peptide content in JHBP-0 was 8.80%, which is basically consistent with previous studies about peptides in Jinhua ham (8.95%) [12] and Xuanwei ham (8.49%) [22].

### 3.3. Peptide Molecular Weight (MW) Distribution

**Figure 1:**
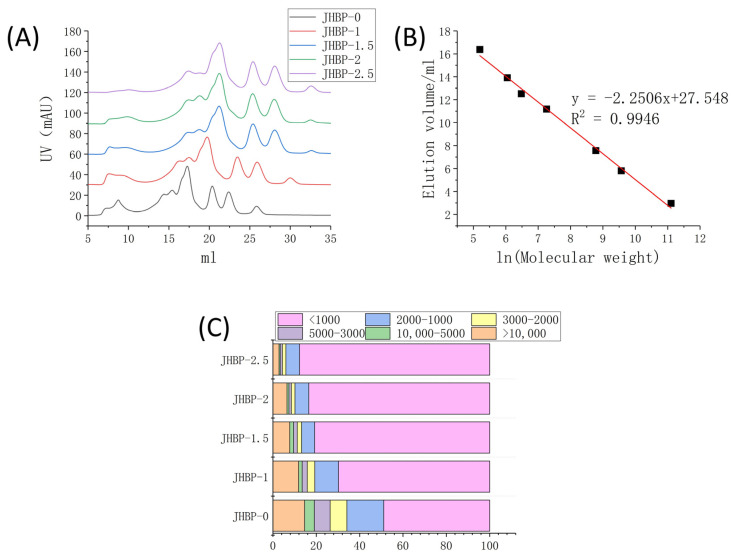
(**A**) UV value of FPLC system. (**B**) Standard curve. (**C**) Stacking diagram of different molecular weights (*n* = 3).

Figure 1A reveals the peptide accumulation at various cooking times. Figure 1C displays the percentage of peptides with different molecular weights calculated from Figure 1B. Compared to JHBP-0, the contents of smaller molecule peptides were increased in the cooking groups, and the fraction of large molecule peptides gradually declined as the cooking duration increased, which changed the mass distribution of the peptides retrieved from the broth. The proportion of peptides < 1 kDa was significantly increased (*p* < 0.05) in all four cooked groups, and the proportion increased from 48.88% at JHBP-0 to 87.74% after 2.5 h of cooking. The fraction of peptides in the mass ranged from 1–2 kDa, 2–3 kDa, 3–5 kDa and >10 kDa dropped steadily (*p* < 0.05). The proportion of 5–10 kDa peptides was without significant differences (*p* > 0.05) between cooking groups.

Previous studies have shown that high-temperature heating can cause the fracture of myofibrils [23] and collagen fibers [24] in meat and give rise to significant changes in ultrastructure. The Z-lines of myofibrils were broken and the M-lines became blurred. Furthermore, the appearance of collagen fibers was obscure, swollen, and fragmented severely. The solubility of myofibrillar proteins and collagen proteins also increased. The degradation of protein and the increase of solubility led to a considerable number of newly produced small molecular proteins and peptides being dissolved in the broth. Sasaki et al. [20] reported that enormous amounts of oligopeptides were released during the cooking of pork, and the longer the cooking time, the more amounts of oligopeptides released. The amount of oligopeptide reached the maximum at 180 min of cooking. After cooking, the peptides extracted from the longissimus dorsi of pigs had antioxidant and antihypertensive activities [25]. The above results indicate that cooking can increase the proportion of small molecular peptides, which is consistent with the results of this current study. The gradual increase of small molecular peptides also improved the potential biological activity of broth.

### 3.4. Antioxidation Activity of JHBP

**Figure 2:**
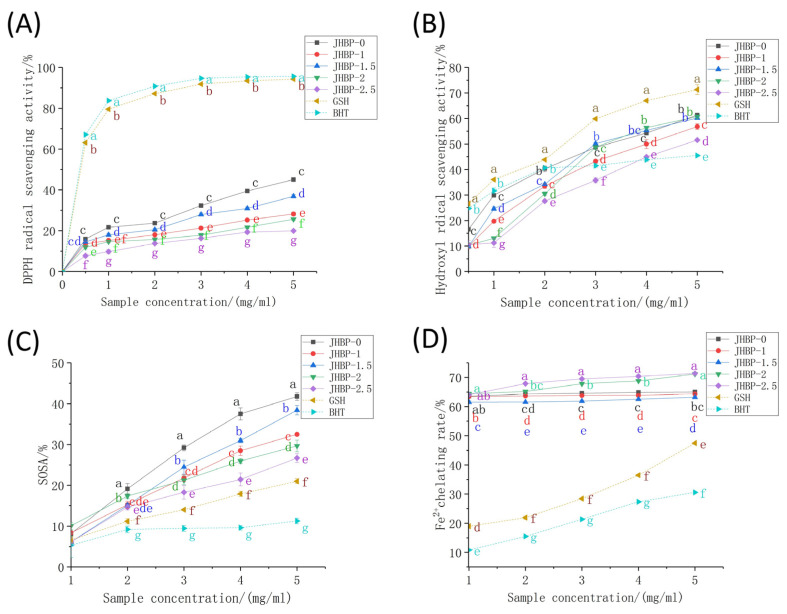
Antioxidant capacity: (**A**) DPPH radical scavenging activity. (**B**) Hydroxyl radical scavenging activity. (**C**) Superoxide anion radical scavenging activity. (**D**) Fe^2+^ chelating activity. Different letters (a–g) represent significant differences (*p* < 0.05, *n* = 5).

The antioxidant capacity of peptides was tested utilizing six different methods in the current study at various cooking times (Figure 2, Table 3). Regardless of whether cooked or not, the DPPH radical scavenging rates of ham-derived peptides (Figure 2A) were noticeably lower than those of GSH and BHT (*p* < 0.05). DPPH scavenging effect increased gradually as peptide concentrations increased, where the activity of the JHBP-0 (45.04%) and JHBP-1.5 (35.84%) were stronger, likened to the other groups (*p* < 0.05). The lowest antioxidant activity was found at 2.5 h of cooking, with a trend of antioxidant activity firstly rising and then falling as cooking time increased. Previous studies have shown that cooking reduced the oxygen radical absorbance capacity (ORAC) and DPPH radical scavenging activity of the peptide in pork, beef, and eggs [26,27]. These findings align with our current results which indicated that long-time cooking reduced the DPPH radical scavenging activity.

For the hydroxyl radical (•OH) testing, the clearance rates of peptide were dose-dependent (>1 mg/mL) and increased rapidly with the increase of concentration (Figure 2B). In contrast, the clearance rates of the BHT increased slowly when the concentration raised higher than 2 mg/mL. The clearance rate of the JHBP-0 was significantly higher than the four boiled groups (*p* < 0.05). The JHBP-0 and the BHT had no significant differences at 1.5 mg/mL (*p* > 0.05), which were higher compared to the four boiling groups (*p* < 0.05). Except for JHBP-2.5, the other four groups considerably surpassed the BHT (*p* < 0.05) at the 3 mg/mL concentration level. Besides, no significant difference was detected between JHBP-0, JHBP-1.5, and JHBP-2 at 5 mg/mL (*p* > 0.05), which had a higher scavenging rate than JHBP-1 (*p* < 0.05). At 5 mg/mL, the clearance rate of JHBP-2.5 was higher compared to the BHT group (*p* < 0.05) while being the lowest among the five groups. At each concentration level, the •OH scavenging effect of the five treatment groups was lower significantly compared to the GSH (*p* < 0.05). In Table 3, the JHBP-0 (29.58 μmol Fe^2+^/g) had a higher FRAP value than the other four cooking groups. The JHBP-1.5 (25.06 μmol Fe^2+^/g)—which was the highest among the four groups—had no significant differences from the JHBP-0 value (*p* > 0.05), and JHBP-1 had the lowest FRAP value. The FRAP value initially increased and then decreased when the cooking time was extended. Additionally, cooking time significantly increased the ABTS^+^ radical scavenging ability (*p* < 0.05), which was stronger compared to the control group (*p* < 0.05).

The SOSA actions of peptides, GSH and BHT were all in the range of 5–10% (Figure 2C). The scavenging rates of peptides were more significant than GSH and BHT (*p* < 0.05) at a 1 mg/mL concentration increment. Also, the Fe^2+^ chelating activity was significantly stronger among the five treatment groups compared to GSH and BHT in the range of 1–5 mg/mL, which was related to the presence of FAAs in the treatment groups (Figure 2D). JHBP-2 and JHBP-2.5 possessed higher Fe^2+^ chelation rates, reaching 71.16% and 71.37%, respectively. One potential explanation is that the peptide produced by long-time cooking exposed more polar groups (acid groups or basic groups), making it easier to combine with Fe^2+^ to form a more stable chelating structure [10].

The current methods used to determine in vitro antioxidant capacity are assays based on hydrogen atom transfer (HAT) reactions or electron transfer (ET) [28]. These two types of reactions assess the antioxidant capacity of antioxidants in terms of their ability to provide hydrogen ions and electrons to scavenge free radicals. The determination based on the HAT reaction is more often used for the determination of the antioxidant capacity of lipophilic antioxidants (such as vitamin E and β-carotene) [29], and the determination conditions are contradictory to the actual conditions of food systems [28]. Therefore, the DPPH radical scavenging activity and T-AOC (ABTS radical scavenging test and ferric reducing antioxidant capacity) used in this study are ET-based assays.

The molecular weight of peptides with a strong antioxidant capacity was generally 400–2000 Da [1]. The results of the FPLC system showed that cooking significantly increased the proportion of <2 KDa peptides. The polarity of proteins and peptides showed a negative correlation with hydrophobicity [30]. The breakdown of peptide bonds could release highly hydrophilic polar chemical groups (amino and carboxyl) [31], which lead to a gradual decrease in the hydrophobicity of Jinhua ham broth peptides during cooking. This made it more difficult for the relatively lipophilic DPPH radical to approach the peptide and easier for the relatively hydrophilic ABTS^+^ radical to approach the peptide. According to the ET reaction principle, when the peptide delivers electrons, the difficulty of binding the two radicals to the electrons varies depending on the distance from the peptide, which may be the main reason for the different results obtained from these two antioxidant experiments.

Cooking increases peptides’ free amino acid content, which has a dual role in peptide biological activity. Amino acids and peptides have synergistic antioxidant effects [32]. Depending on the partial least squares (PLS) regression, Udenigwe et al. [33] investigated the contribution of independent amino acid residues or combinations of amino acids to the antioxidant activity of some food protein hydrolysates (flaxseed protein, pea seed protein, ferritin, lactalbumin, etc.). They reported that food protein hydrolysates constituting moderate amounts of FAA had effective antioxidant attributes. Hwang et al. [34] and Zhang et al. [35] argued that the presence of FAA in soybean oil and vinegar was helpful to boost their antioxidant capacity.

Additionally, cooking prompted the breakdown of peptides chain and generated novel FAA, which may reduce their biological activity. Even though the amino acids of Tyr, Met, His, Lys, and Trp have antioxidant activities when presented as FAA, studies have shown that dipeptides (including Ala, Tyr, His, or Met) had better antioxidant activity in aqueous systems than mixes of constituent amino acids [36]. Blanca et al. [37] isolated 42 antioxidant peptides from β-lactoglobulin and discovered that the antioxidation capacity of the Trp-Tyr-Ser-Leu-Ala-Met-Ala-Ala-Ser-Ile was lower compared to the equimolar amino acid solution. In contrast, the antioxidation efficacy of the Tyr-Val-Glu-Glu-Leu sequence was higher compared to the equimolar amino acid mixture. The peptide bond or peptide conformation may be related to the short-range interactions between amino acids inside the peptide chain, which may have antagonistic or synergistic effects on their antioxidant properties.

### 3.5. Secondary Structure of Peptides

**Figure 3:**
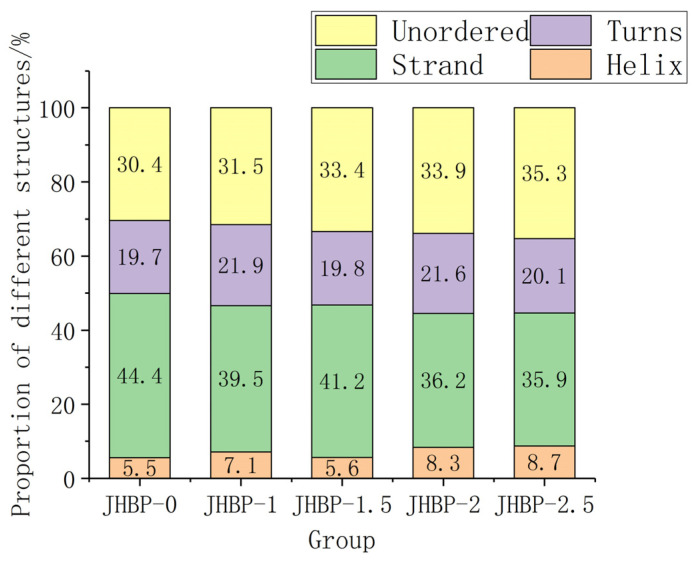
The proportion of secondary structures (*n* = 3).

Previous studies have shown that low α-helix and high β-structure (β-strand and β-turns) possibly favored peptide to exert antioxidation action [38]. Figure 3 depicts the fraction of secondary structure occupied in water by helix, strand, turns, and unordered. In aqueous form, JHBP-0 had the highest strand ratio (44.4%) and the lowest helix ratio (5.5%). This is consistent with the result that JHBP-0 showed the strongest antioxidant capacity. JHBP-1.5 showed a lower helix ratio (5.6%) and a higher strand ratio (41.2%) than those of the other three cooked groups. Similarly, the antioxidant capacity of JHBP-1.5 was slightly stronger than the other three cooked groups. Compared with JHBP-0 (30.4%), all four cooking groups had higher unordered ratios (31.5–35.3%), suggesting that cooking led the peptide to exhibit a more disordered secondary structure.

### 3.6. Peptidomics

**Figure 4:**
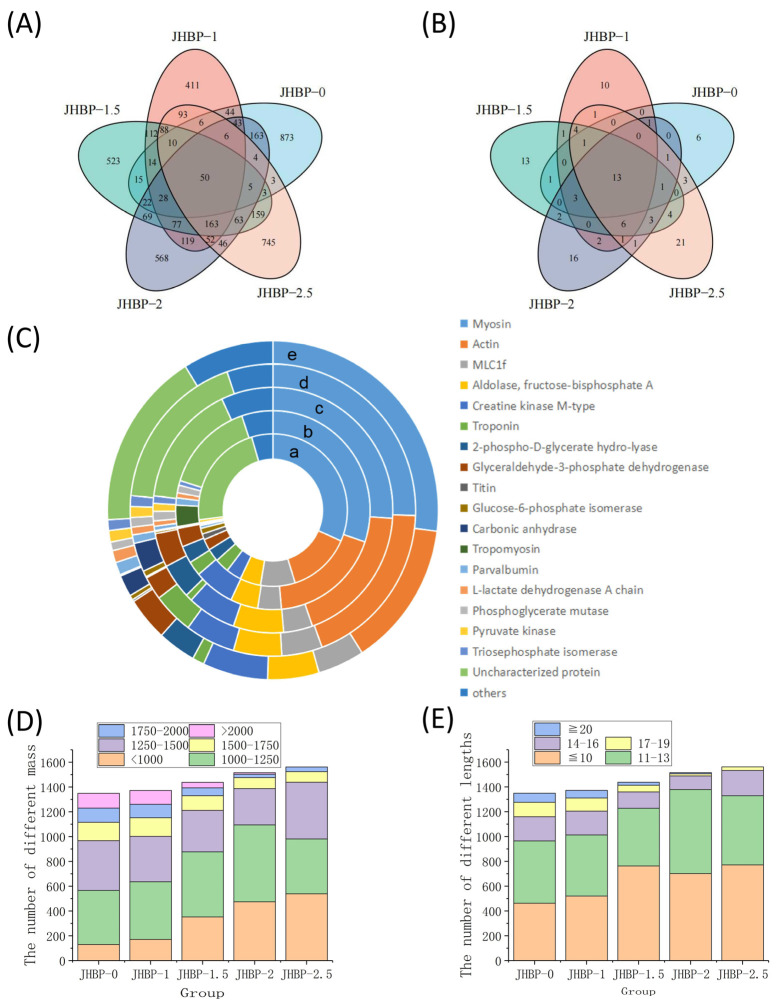
(**A**) Venn diagram of characteristic peptides. (**B**) Source proteins for peptides. (**C**) Distribution in percentages of peptides according to the origin proteins in the muscle of Sus scrofa: a. JHBP-0; b. JHBP-1; c. JHBP-1.5; d. JHBP-2; e. JHBP-2.5. (**D**) The number of different masses. (**E**) The number of different lengths (*n* = 3).

There were 1306, 1352, 1431, 1500, and 1556 peptide sequences found for JHBP-0, JHBP-1, JHBP-1.5, JHBP-2, and JHBP-2.5, respectively (Figure 4A). The 50 of these peptides were repeated in the five groups, while the unique peptide sequences were 873, 411, 523, 567, and 745 for each group, respectively. Unique peptide sequences in JBHP-0 outnumbered other groups, while JBHP-1 had the least unique peptide sequences. The variety of unique peptide sequences gradually increased with the increase in cooking time. Cooking significantly influenced the composition of peptides, with a loss of the peptides in raw ham. Likewise, many novel peptides were generated during the cooking process. Hence, there were 13 identical source proteins in the five groups of which 6, 10, 13, 16, and 20 source proteins were unique to the five groups, respectively (Figure 4B). The number of distinctive source proteins was dependent on the cooking time process. In identical source proteins, myosin and actin were the most prevalent sources of peptides (Figure 4C). This is consistent with earlier investigations by the proteomics homology of antioxidant peptides isolated from Jinhua and Xuanwei ham [12]. The number of peptides from the four source proteins, including myosin, actin, mLC1f and aldolase-fructose-diphosphate A, accounted for more than 50% in each group.

The peptide segments were analyzed according to the peptide mass (Figure 4D) and lengths (Figure 4E). The trend of mass change is similar to the results obtained from the previously measured mass distribution. There were only 105 peptides smaller than 1000 Da in JHBP-0 and 381 peptides larger than 1500 Da, including 119 peptides larger than 2000 Da. In contrast, cooking produced more small molecule peptides, with 536 peptides less than 1000 Da in JHBP-2.5, only 123 peptides greater than 1500 Da, and no detectable ones greater than 2000 Da. From the perspective of peptide chain length analysis, with the increase of cooking time, the peptides of large molecules decomposed, and the detection of peptides with longer peptide chains of more than 20 amino acids gradually decreased, which was not detected in JHBP-2.5. Meanwhile, the peptides, in the range of 1250–1500 Da and 14–16 AA, gradually reduced with increased cooking time until 2 h. However, this part of JHBP-2.5 increased relative to JHBP-2, presumably because the prolonged heating time caused some proteins to break down, thus they could not be easily decomposed in the dry-cured ham before cooking.

## 4. Conclusions

The current research revealed that different cooking times altered the antioxidant and peptide profile of Jinhua ham broth peptides. The antioxidant capacity of peptides is related to the content of FAA, secondary structure, molecular weight, and peptide sequence. The FAA content of crude peptides increased with cooking time, and the concentrations of Lys, Glu, Ala, Leu, Arg, and Val were much higher than other AAs in all five experimental groups. CD spectroscopy results indicated that prolonged cooking led to a more disordered peptide secondary structure as the β-structure/α-helix ratio decreased. Meanwhile, the results of the FPLC system showed that cooking significantly increased the proportion of <1 KDa peptides and significantly reduced the proportion of peptides in all molecular weight ranges except for 5–10 kDa peptides. LC-MS/MS results validated that cooking increased the number of peptide sequences and source proteins, as well as the abundance of peptides released from myosin and actin, thereby further demonstrating that cooking promoted proteolysis. All the above results affected the antioxidant activity of the Jinhua ham broth peptide. In the cooked groups, JHBP-1.5 showed stronger antioxidant activities for DPPH, •OH, SOSA, and FRAP radical scavenging efficacy, as well as stronger antioxidant activity of JHBP-2.5 for Fe^2+^ chelation capacity and ABTS^+^ scavenging capacity. Overall, our study uncovered the rich bioactive substances in Jinhua ham broth, and highlighted its potential bioactivity for human health when included in traditional Chinese dishes.

## Figures and Tables

**Table 1:** Free amino acids of the JHBP.

	JHBP-0/ (mg/50 g ham)	JHBP-1/ (mg/100 mL Broth)	JHBP-1.5/ (mg/100 mL Broth)	JHBP-2/ (mg/100 mL Broth)	JHBP-2.5/ (mg/100 mL Broth)
Asp	26.71 ± 0.36 ^Df^	33.07 ± 0.39 ^Cf^	35.73 ± 0.85 ^Cf^	57.82 ± 3.00 ^Bf^	65.85 ± 0.76 ^Af^
Thr	23.50 ± 0.39 ^Dh^	28.71 ± 0.40 ^Ch^	30.14 ± 0.68 ^Ch^	48.65 ± 1.86 ^Bg^	56.30 ± 0.65 ^Ah^
Ser	25.63 ± 0.51 ^Bfg^	31.01 ± 0.31 ^Bg^	32.69 ± 1.20 ^Bg^	57.42 ± 7.42 ^Af^	61.38 ± 0.93 ^Ag^
Glu	67.43 ± 0.78 ^Eb^	83.88 ± 1.33 ^Db^	88.89 ± 2.05 ^Cb^	138.02 ± 0.97 ^Bb^	162.95 ± 2.40 ^Ab^
Gly	16.06 ± 0.42 ^Dj^	19.46 ± 0.26 ^Bk^	20.16 ± 0.67 ^Bk^	34.21 ± 3.20 ^Akj^	37.60 ± 0.49 ^Ak^
Ala	45.16 ± 0.67 ^Dc^	54.89 ± 1.34 ^Cc^	57.48 ± 1.36 ^Cc^	91.03 ± 2.96 ^Bc^	107.15 ± 1.51 ^Ac^
Val	32.48 ± 0.49 ^De^	38.11 ± 0.70 ^Ce^	40.21 ± 1.00 ^Ce^	63.50 ± 1.86 ^Be^	75.63 ± 1.62 ^Ae^
Met	13.15 ± 0.57 ^Dk^	15.35 ± 0.07 ^Cm^	15.89 ± 0.24 ^Cl^	25.26 ± 0.57 ^Bl^	30.46 ± 0.37 ^Al^
Ile	21.61 ± 0.34 ^Di^	24.38 ± 0.47 ^Cj^	25.61 ± 0.12 ^Ci^	41.36 ± 1.50 ^Bi^	49.88 ± 0.86 ^Ai^
Leu	40.91 ± 0.79 ^Dd^	46.18 ± 0.60 ^Cd^	48.44 ± 0.27 ^Cd^	78.62 ± 2.06 ^Bd^	94.38 ± 1.84 ^Ad^
Tyr	15.35 ± 0.90 ^Ej^	19.73 ± 0.21 ^Dk^	22.67 ± 0.49 ^Cj^	35.88 ± 0.86 ^Bj^	43.79 ± 1.23 ^Aj^
Phe	24.02 ± 1.52 ^Dgh^	28.63 ± 0.55 ^Ch^	29.49 ± 1.67 ^Ch^	47.51 ± 0.79 ^Bgh^	57.22 ± 0.81 ^Ah^
Lys	70.67 ± 1.34 ^Da^	94.26 ± 1.14 ^Ca^	96.67 ± 1.44 ^Ca^	152.49 ± 1.88 ^Ba^	190.77 ± 4.52 ^Aa^
His	13.52 ± 0.30 ^Dk^	17.77 ± 0.11 ^Cl^	18.13 ± 0.13 ^Ck^	30.39 ± 1.60 ^Bkl^	36.28 ± 0.96 ^Ak^
Arg	33.74 ± 1.35 ^De^	45.12 ± 0.33 ^Cd^	46.63 ± 0.76 ^Cd^	74.09 ± 1.32 ^Bd^	94.25 ± 3.09 ^Ad^
Pro	21.61 ± 0.62 ^Di^	26.79 ± 0.89 ^Ci^	27.41 ± 0.45 ^Ci^	42.52 ± 1.48 ^Bhi^	49.98 ± 0.63 ^Ai^
Total	491.54 ± 4.96 ^D^	607.33 ± 7.05 ^C^	636.22 ± 6.32 ^C^	1018.76 ± 18.98 ^B^	1213.86 ± 20.20 ^A^

Note: (1) A–E superscripts indicate significant differences in each row (*p* < 0.05, *n* = 3), (2) a–m superscripts indicate significant differences in each column (*p* < 0.05, *n* = 3).

**Table 2:** Extraction of polypeptide from Jinhua ham broth at different cooking times.

Group	Crude Extracts g/100 g ham	Peptide Content in Crude Extracts/%	Peptide Extraction/%
JHBP-0	9.77 ± 0.88 ^d^	89.99 ± 0.65 ^a^	8.80 ± 0.80 ^d^
JHBP-1	12.19 ± 0.30 ^c^	87.59 ± 0.97 ^b^	10.67 ± 0.24 ^c^
JHBP-1.5	14.33 ± 0.33 ^b^	87.21 ± 1.28 ^b^	12.50 ± 0.15 ^b^
JHBP-2	15.66 ± 041 ^a^	86.71 ± 1.38 ^b^	13.58 ± 0.19 ^a^
JHBP-2.5	15.94 ± 0.46 ^a^	86.87 ± 0.90 ^b^	13.85 ± 0.28 ^a^

Note: a–d superscripts indicate significant differences in each column (*p* < 0.05, *n* = 6).

**Table 3:** Total antioxidant capacity of JHBP.

Group	FRAP (μmol Fe^2+^/g)	ABTS^+^ (μmol TE/g)
JHBP-0	29.58 ± 3.01 ^a^	261.88 ± 4.22 ^e^
JHBP-1	19.18 ± 1.00 ^c^	272.74 ± 0.68 ^d^
JHBP-1.5	25.06 ± 2.91 ^ab^	317.99 ± 4.05 ^c^
JHBP-2	22.09 ± 3.79 ^bc^	374.05 ± 3.44 ^b^
JHBP-2.5	20.30 ± 1.02 ^c^	390.02 ± 0.53 ^a^

Note: a–e superscripts indicate significant differences in each column (*p* < 0.05, *n* = 5).

## Data Availability

Data is contained within the article.

## References

[1] Toldra F., Gallego M., Reig M., Aristoy M., Mora L. (2020). Bioactive peptides generated in the processing of dry-cured ham. Food Chem..

[2] Zhang J., Zhen Z., Zhang W., Zeng T., Zhou G. (2009). Effect of intensifying high-temperature ripening on proteolysis, lipolysis and flavor of Jinhua ham. J. Sci. Food Agric..

[3] Yin Y., Pereira J., Zhou L., Lorenzo J.M., Tian X., Zhang W. (2020). Insight into the effects of sous vide on cathepsin B and L activities, protein degradation and the ultrastructure of beef. Foods.

[4] Gallego M., Mora L., Toldra F. (2019). The relevance of dipeptides and tripeptides in the bioactivity and taste of dry-cured ham. Food Prod. Process. Nutr..

[5] Liu R., Xing L., Fu Q., Zhou G., Zhang W. (2016). A review of antioxidant peptides derived from meat muscle and by-products. Antioxidants.

[6] Xing L., Liu R., Cao S., Zhang W., Zhou G. (2019). Meat protein based bioactive peptides and their potential functional activity: A review. Int. J. Food Sci. Technol..

[7] Zhang W., Naveena B.M., Jo C., Sakata R., Zhou G., Banerjee R., Nishiumi T. (2017). Technological demands of meat processing-an asian perspective. Meat Sci..

[8] Wang Z. (2017). Chinese Medicinal Diet Dictionary.

[9] Dai Y., Zhang Q., Wang L., Liu Y., Li X., Dai R. (2014). Changes in shear parameters, protein degradation and ultrastructure of pork following water bath and ohmic cooking. Food Bioprocess Technol..

[10] Zhu C., Zhang W., Kang Z., Zhou G., Xu X. (2014). Stability of an antioxidant peptide extracted from Jinhua ham. Meat Sci..

[11] Xing L., Hu Y., Hu H., Ge Q., Zhou G., Zhang W. (2016). Purification and identification of antioxidative peptides from dry-cured Xuanwei ham. Food Chem..

[12] Xing L., Liu R., Gao X., Zheng J., Wang C., Zhou G., Zhang W. (2018). The proteomics homology of antioxidant peptides extracted from dry-cured Xuanwei and Jinhua ham. Food Chem..

[13] Xing L., Fu L., Toldra F., Teng S., Yin Y., Zhang W. (2022). The stability of dry-cured ham-derived peptides and its anti-inflammatory effect in RAW264.7 macrophage cells. Int. J. Food Sci. Technol..

[14] Erickson M.C. (1993). Lipid extraction from channel catfish muscle-comparison of solvent systems. J. Food Sci.

[15] Zhang J., Li M., Zhang G., Tian Y., Kong F., Xiong S., Zhao S., Jia D., Manyande A., Du H. (2021). Identification of novel antioxidant peptides from snakehead (*Channa argus*) soup generated during gastrointestinal digestion and insights into the anti-oxidation mechanisms. Food Chem..

[16] Zhao D., Li L., Le T.T., Larsen L.B., Su G., Liang Y., Li B. (2017). Digestibility of glyoxal-glycated beta-casein and beta-lactoglobulin and distribution of peptide-bound advanced glycation end products in gastrointestinal digests. J. Agric. Food Chem..

[17] Chen M., Ning P., Jiao Y., Xu Z., Cheng Y. (2021). Extraction of antioxidant peptides from rice dreg protein hydrolysate via an angling method. Food Chem..

[18] Yu D., Feng M., Sun J. (2021). Influence of mixed starters on the degradation of proteins and the formation of peptides with antioxidant activities in dry fermented sausages. Food Control.

[19] Rotola-Pukkila M.K., Pihlajaviita S.T., Kaimainen M.T., Hopia A.I. (2015). Concentration of umami compounds in pork meat and cooking juice with different cooking times and temperatures. J. Food Sci..

[20] Sasaki K., Motoyama M., Mitsumoto M. (2007). Changes in the amounts of water-soluble umami-related substances in porcine longissimus and biceps femoris muscles during moist heat cooking. Meat Sci..

[21] Zhu C., Tian W., Sun L., Liu Y., Li M., Zhao G. (2019). Characterization of protein changes and development of flavor components induced by thermal modulation during the cooking of chicken meat. J. Food Process. Preserv..

[22] Fu L., Xing L., Hao Y., Yang Z., Teng S., Wei L., Zhang W. (2021). The anti-inflammatory effects of dry-cured ham derived peptides in RAW264.7 macrophage cells. J. Funct. Foods.

[23] Song Y., Huang F., Li X., Han D., Zhang C. (2021). Effects of different wet heating methods on the water distribution, microstructure and protein denaturation of pork steaks. Int. J. Food Sci. Technol..

[24] Lin Y., Wang Y., Jin G., Duan J., Zhang Y., Cao J. (2022). The texture change of chinese traditional pig trotter with soy sauce during stewing processing: Based on a thermal degradation model of collagen fibers. Foods.

[25] Simonetti A., Gambacorta E., Perna A. (2016). Antioxidative and antihypertensive activities of pig meat before and after cooking and in vitro gastrointestinal digestion: Comparison between Italian autochthonous pig Suino Nero Lucano and a modern crossbred pig. Food Chem..

[26] Jensen I., Dort J., Eilertsen K. (2014). Proximate composition, antihypertensive and antioxidative properties of the semimembranosus muscle from pork and beef after cooking and in vitro digestion. Meat Sci..

[27] Remanan M.K., Wu J. (2014). Antioxidant activity in cooked and simulated digested eggs. Food Funct..

[28] Huang D.J., Ou B.X., Prior R.L. (2005). The chemistry behind antioxidant capacity assays. J. Agric. Food Chem..

[29] Han R., Cheng H., Feng R., Li D., Lai W., Zhang J., Skibsted L.H. (2014). beta-Carotene As a Lipophilic Scavenger of Nitric Oxide. J. Phys. Chem B.

[30] Schmid S., Adjobo-Hermans M.J.W., Kohze R., Enderle T., Brock R., Milletti F. (2017). Identification of short hydrophobic cell-penetrating peptides for cytosolic peptide delivery by rational design. Bioconjug. Chem..

[31] Lacou L., Leonil J., Gagnaire V. (2016). Functional properties of peptides: From single peptide solutions to a mixture of peptides in food products. Food Hydrocoll..

[32] Liu J., Zhang D., Zhu Y., Wang Y., He S., Zhang T. (2018). Enhancing the in vitro antioxidant capacities via the interaction of amino acids. Emir. J. Food Agric..

[33] Udenigwe C.C., Aluko R.E. (2011). Chemometric analysis of the amino acid requirements of antioxidant food protein hydrolysates. Int. J. Mol. Sci..

[34] Hwang H., Winkler-Moser J.K. (2017). Antioxidant activity of amino acids in soybean oil at frying temperature: Structural effects and synergism with tocopherols. Food Chem..

[35] Zhang B., Xia T., Duan W., Zhang Z., Li Y., Fang B., Xia M., Wang M. (2019). Effects of organic acids, amino acids and phenolic compounds on antioxidant characteristic of zhenjiang aromatic vinegar. Molecules.

[36] Chen H.M., Muramoto K., Yamauchi F., Nokihara K. (1996). Antioxidant activity of designed peptides based on the antioxidative peptide isolated from digests of a soybean protein. J. Agric. Food Chem..

[37] Hernandez-Ledesma B., Davalos A., Bartolome B., Amigo L. (2005). Preparation of antioxidant enzymatic hydrolysates from (alpha-lactalbumin and beta-lactoglobulin. Identification of active peptides by HPLC-MS/MS. J. Agric. Food Chem..

[38] Yuan H., Lv J., Gong J., Xiao G., Zhu R., Li L., Qiu J. (2018). Secondary structures and their effects on antioxidant capacity of antioxidant peptides in yogurt. Int. J. Food Prop..

